# Poly(I:C) increases the expression of mPGES-1 and COX-2 in rat primary microglia

**DOI:** 10.1186/s12974-015-0473-7

**Published:** 2016-01-18

**Authors:** Antonio Carlos Pinheiro de Oliveira, Nizar M. Yousif, Harsharan Singh Bhatia, Julia Hermanek, Michael Huell, Bernd L. Fiebich

**Affiliations:** Department of Pharmacology, Institute of Biological Sciences, Universidade Federal de Minas Gerais, Av. Antonio Carlos 6627, 31270-901 Belo Horizonte, MG Brazil; Department of Psychiatry, University of Freiburg Medical School, Hauptstr. 5, 79104 Freiburg, Germany; Zentrum für Geriatrie und Gerontologie, Sektion Gerontopsychiatrie und Neuropsychologie, Universitätsklinikums Freiburg, Lehener Str. 88, 79106 Freiburg, Germany; Clinic for Geriatric Psychiatry, Center for Psychiatry Emmendingen, Neubronnstraße 25, 79312 Emmendingen, Germany; VivaCell Biotechnology GmbH, Ferdinand-Porsche-Str. 5, 79211 Denzlingen, Germany

**Keywords:** Polyinosinic-polycytidylic acid, TLR3, COX-2, mPGES-1, Microglia

## Abstract

**Background:**

Microglia recognize pathogen-associated molecular patterns such as double-stranded RNA (dsRNA) present in some viruses. Polyinosinic-polycytidylic acid [poly(I:C)] is a synthetic analog of dsRNA that activates different molecules, such as retinoic acid-inducible gene I, melanoma differentiation-associated gene 5, and toll-like receptor-3 (TLR3). Poly(I:C) increases the expression of different cytokines in various cell types. However, its role in the regulation of the production of inflammatory mediators of the arachidonic acid pathway by microglia is poorly understood.

**Methods:**

In the present study, we evaluated the effect of poly(I:C) on the production of prostaglandin E_2_ (PGE_2_) and the inducible enzymes cyclooxygenase-2 (COX-2) and microsomal prostaglandin E synthase-1 (mPGES-1) in primary rat microglia. Microglia were stimulated with different concentrations of poly(I:C) (0.1–10 μg/ml), and the protein levels of COX-2 and mPGES-1, as well as the release of PGE_2_, were determined by western blot and enzyme immunoassay (EIA), respectively. Values were compared using one-way ANOVA with post hoc Student-Newman-Keuls test.

**Results:**

Poly(I:C) increased the production of PGE_2_, as well as mPGES-1 and COX-2 synthesis. To investigate the mechanisms involved in poly(I:C)-induced COX-2 and mPGES-1, we studied the effects of various signal transduction pathway inhibitors. Protein levels of COX-2 and mPGES-1 were reduced by SB203580, SP600125, and SC514 (p38 mitogen-activated protein kinase (MAPK), c-Jun N-terminal kinase (JNK), and IκB kinase (IKK) inhibitors, respectively), as well as by PD98059 and PD0325901 (mitogen-activated protein kinase kinase (MEK) inhibitors). Rapamycin, a mammalian target of rapamycin (mTOR) inhibitor, enhanced the synthesis of COX-2. Inhibition of phosphatidylinositol 3-kinase (PI3K) by LY294002 or dual inhibition of PI3K/mTOR (with NVP-BEZ235) enhanced COX-2 and reduced mPGES-1 immunoreactivity. To confirm the data obtained with the inhibitors, we studied the phosphorylation of the blocked kinases by western blot. Poly(I:C) increased the phosphorylation of p38 MAPK, extracellular signal-regulated kinase (ERK), JNK, protein kinase B (Akt), and IκB.

**Conclusions:**

Taken together, our data demonstrate that poly(I:C) increases the synthesis of enzymes involved in PGE_2_ synthesis via activation of different signaling pathways in microglia. Importantly, poly(I:C) activates similar pathways also involved in TLR4 signaling that are important for COX-2 and mPGES-1 synthesis. Thus, these two enzymes and their products might contribute to the neuropathological effects induced in response to dsRNA, whereby the engagement of TLR3 might be involved.

## Background

Microglia express receptors that recognize pathogen-associated molecular patterns leading to various immunological responses. For instance, the toll-like receptor-3 (TLR3) is an intracellular receptor that recognizes double-stranded RNA produced by some viruses [1, 2]. TLR3 might also be activated by endogenous messenger RNA (mRNA) released from apoptotic cells [3], and activation of this receptor has been shown to increase the expression of different cytokines in various cell types, including microglia [4, 5].

Polyinosinic-polycytidylic acid [poly(I:C)] is a ligand of TLR3 and is able to activate different immune cells in a toll/interleukin (IL)-1 receptor domain-containing adaptor inducing IFN-β (TRIF)-dependent manner [6]. Activation of this receptor is important to protect and fight against infections, since its activation induces the production of type I interferon and other cytokines involved in antiviral responses [7]. Furthermore, poly(I:C) increases the phagocytosis and the intracellular killing of *Escherichia coli* by primary microglia [8].

Besides its role in infections, TLR3 activation might be involved in neurodegeneration, psychiatric disorders, and pain [2, 9–12]. Considering that RNA released from necrotic cells could activate TLR3 [3], it may be assumed that the binding of endogenous nucleic acid released from dying neurons could activate TLR3 in other cell types, such as microglia, and promote an inflammatory process in the brain. Systemic administration of poly(I:C) increases apoptosis and exacerbates an existing chronic neurodegenerative process in a ME7 model of prion disease [9]. Furthermore, injection of poly(I:C) enhances neuronal loss in the substantia nigra pars compacta and striatum induced by 6-hydroxydopamine and paraquat [13, 14]. Additionally, challenge of mice aged 5 to 7 days with poly(I:C) induces schizophrenia-like signs, as well as a progressive microglia activation [15]. Indeed, prenatal injection of poly(I:C) in rodents is used as a neurodevelopmental model of schizophrenia [2, 16].

Although different studies have demonstrated that the effects of poly(I:C) might be dependent on TLR3, it is currently known that this compound acts via other targets. To date, it has been shown that poly(I:C) activates retinoic acid-inducible gene I (RIG-I) and melanoma differentiation-associated gene 5 (MDA5), which are also pattern recognition receptors (PRRs) that recognize pathogen-specific molecular patterns [17, 18]. Interestingly, the involvement of these PRRs in neurodegeneration has also been suggested [19, 20].

Although the pathological conditions induced by poly(I:C) might be a consequence of an inflammatory process that leads to neurodevelopmental abnormalities, neurodegenerative processes, or pain, the underlying mechanisms are still unknown. These effects might be associated with microglia activation [21], which results in the release of neurotoxic molecules such as the lipid inflammatory mediators from the arachidonic acid cascade. Since cyclooxygenase-2 (COX-2), microsomal prostaglandin E synthase-1 (mPGES-1), and prostaglandin E_2_ (PGE_2_) are involved in neurodegeneration, psychiatric disorders, and pain [22–26], these molecules may mediate the pathological effects induced by dsRNA.

Thus, it is necessary to unveil molecular mechanisms induced by a viral mimetic in isolated brain microglial cells, since these cells are the main source of various inflammatory mediators. Different studies use lipopolysaccharide (LPS) as a gold standard to activate microglia, but the main receptor of this substance is the TLR4. However, although it has been shown that TLR3 ligands increase the production of cytokines in microglia [27, 28], the role of this receptor in the production of inflammatory lipid mediators in microglia is poorly understood. In the present study, we evaluated the effect of poly(I:C) in the synthesis of molecules involved in the arachidonic acid cascade (i.e., COX-2, mPGES-1, and PGE_2_), as well as the intracellular mechanisms involved in these responses in rat primary microglia.

## Methods

The following inhibitors were purchased from Calbiochem (Bad Soden, Germany): PD 98059 (2′-amino-3′-methoxyflavone), an inhibitor of mitogen-activated protein kinase kinase (MEK); SB 203580 [4-(4-fluorophenyl)-2-(4-methylsulfinylphenyl)-5-(4-pyridyl)1H-imidazole], an inhibitor of p38 mitogen-activated protein kinase (MAPK); SP600125 [anthra(1,9-cd)pyrazol-6(2*H*)-one 1,9-pyrazoloanthrone], an inhibitor of c-Jun N-terminal kinase (JNK); rapamycin [(3S,6R,7E,9R,10R,12R,14S,15E,17E,19E,21S,23S,26R,27R,34aS)-9,10,12,13,14,21,22,23,24,25,26,27,32,33,34,34a-hexadecahydro-9,27-dihydroxy-3-[(1R)-2-[(1S,3R,4R)-4-hydroxy-3-methoxycyclohexyl]-1-methylethyl]-10,21-dimethoxy-6,8,12,14,20,26-hexamethyl-23,27-epoxy-3H-pyrido [2,1-c] [1, 4] oxaazacyclohentriacontine-1,5,11,28,29(4H,6H,31H)-pentone], an inhibitor of mammalian target of rapamycin (mTOR); and SC-514 [5-(thien-3-yl)-3-aminothiophene-2-carboxamide], an IκB kinase 2 (IKK2) inhibitor. The dual PI3K/mTOR inhibitor NVP-BEZ235 was purchased from Axon Medchem BV (Groningen, the Netherlands). The phosphatidylinositol 3-kinase (PI3K) inhibitor LY294002 [2-(4-morpholinyl)-8-phenyl-4H-1-benzopyran-4-one hydrochloride] and the MEK inhibitor PD0325901 [N-[(2R)-2,3-dihydroxypropoxy]-3,4-difluoro-2-[(2-fluoro-4-iodophenyl)amino]-benzamide] were obtained from Tocris (Ellisville, MO). Poly(I:C) (high molecular weight, catalog code: tlrl-pic) was purchased from InvivoGen (San Diego, CA, USA).

All stock solutions were prepared in dimethyl sulfoxide (DMSO) and stored at −20 °C. Further dilutions in DMSO were prepared immediately before the incubations of the cells.

### Primary microglia cultures

Primary microglia cultures were prepared from cerebral cortices of 1-day neonatal Wistar rats [29, 30]. In brief, forebrains were minced and gently dissociated by repeated pipetting in Hank’s balanced salt solution (PAA Laboratories GmbH, Cölbe, Germany). Dissociated cells were then passed through nylon cell strainer with 70-μm pores (BD biosciences, Heidelberg, Germany). Cells were collected by centrifugation, resuspended in Dulbecco’s modified Eagle’s medium (DMEM) containing 10 % fetal calf serum (FCS) and antibiotics and cultured on 10-cm cell culture dishes (Falcon, 5 × 10^5^ cells/plate) in 5 % CO_2_ at 37 °C. Floating microglia were harvested from 12 to 14-day-old mixed (astrocyte-microglia) primary cultures and re-seeded into cell culture plates at the density of 2 × 10^5^ cells/ml to give pure microglial cultures. On the next day, cells were washed to remove non-adherent cells, and fresh medium was added. After 1 h, cells were used for the different experiments.

### Western blot analysis

Thirty minutes, 24 or 48 h after stimulation with poly(I:C), microglial cells were washed with phosphate-buffered saline (PBS) and lysed in 1.3 × SDS (sodium dodecyl sulfate)-containing sample buffer without dithiothreitol (DTT) or bromophenol blue containing 100 μM orthovanadate [31]. Cell lysates were homogenized by repeated passage through a 26-gauge needle. Protein contents were measured using the bicinchoninic acid (BCA) method (Thermo Fischer Scientific, Waltham, MA, USA). Bovine serum albumin (BSA) was used as a protein standard at concentrations ranging from 0.2 to 4 μg/μl, and the optical density was read at 570 nm using a microplate reader. Immediately before electrophoresis, bromophenol blue and DTT (final concentration, 10 mM) were added to the samples. For mPGES-1, COX-2, p-p38 MAPK, p-JNK, p-ERK, p-IκB-α, p-Akt, actin, and α-tubulin immunoblotting, 30 to 50 μg of protein from each sample was subjected to SDS-PAGE (polyacrylamide gel electrophoresis) on a 10 % (for p-p38 MAPK, p-JNK, p-ERK, p-IκB-α, p-Akt) or 12 % (for COX-2 and mPGES-1) gel under reducing conditions. Proteins were then transferred onto a polyvinylidene fluoride (PVDF) membrane (Millipore, Bedford, MA, USA) by semi-dry blotting. The membrane was blocked for 1 or 2 h at room temperature using Rotiblock (Roth, Karlsruhe, Germany) for COX-2 or 5 % blocking milk (BioRad, München, Germany) for the other proteins, before the overnight incubation at 4° C with the primary antibody. Primary antibodies were goat anti-COX-2 (M-19, Santa Cruz, Heidelberg, Germany); rabbit anti-mPGES-1 (Cayman Chemical, USA; 1:500); rabbit anti-actin (Sigma-Aldrich, USA, 1:5000); and rabbit anti-p-p38 anti-MAPK, anti-p-JNK, anti-p-ERK, anti-p-IκB-α, anti-p-Akt, and anti-α-tubulin (all from Cell Signaling Technology). After extensive washing (three times for 15 min each in TBS containing 0.1 % Tween 20), proteins were detected with horseradish peroxidase (HRP)-coupled rabbit anti-goat IgG (Santa Cruz, 1:100,000) or HRP-coupled donkey anti-rabbit (GE Healthcare, 1:25,000) using chemiluminescence (ECL) reagents (GE Healthcare). All western blot experiments were carried out at least three times. The densitometry of the western blot was performed by using Image J software 1.47v (National Institute of Health). A box was drawn around the bands, and a percentage of the area covered was determined. In case of multiple bands, such as phospho-extracellular signal-regulated kinase (ERK) and phospho-JNK where a box was difficult to draw around the single band, quantification of bands was performed altogether as done in our previous study [32]. Highly overexposed bands were excluded in the quantification. The bands of COX-2, mPGES-1, phospho-p38, phospho-ERK, phospho-JNK, phospho-Akt, and phospho-IκB were normalized to actin or α-tubulin as housekeeping proteins.

### Enzyme immunoassay (EIA)

Twenty-four hours after stimulation, supernatants were harvested, centrifuged at 10,000×*g* for 10 min, and levels of PGE_2_ in the media were measured by enzyme immunoassay (EIA) (Biotrend, Köln, Germany) according to the manufacturer’s instructions. Standards from 39 to 2500 pg/ml were used; sensitivity of the assay was 36.2 pg/ml.

### Statistical analysis

At least three independent experiments were used for data analysis. Original data were converted into % values of LPS or poly(I:C) controls, and mean ± S.E.M. were calculated. Values were compared using *t* test (two groups) or one-way ANOVA with post hoc Student-Newman-Keuls test (multiple comparisons). For each experiment, *P* value <0.05 was considered statistically significant. Significant effects are indicated by asterisks (*P < 0.05, **P < 0.01, ***P < 0.001).

## Results

### Poly(I:C) increases the expression of mPGES-1 and COX-2 and PGE_2_ production

**Fig. 1:**
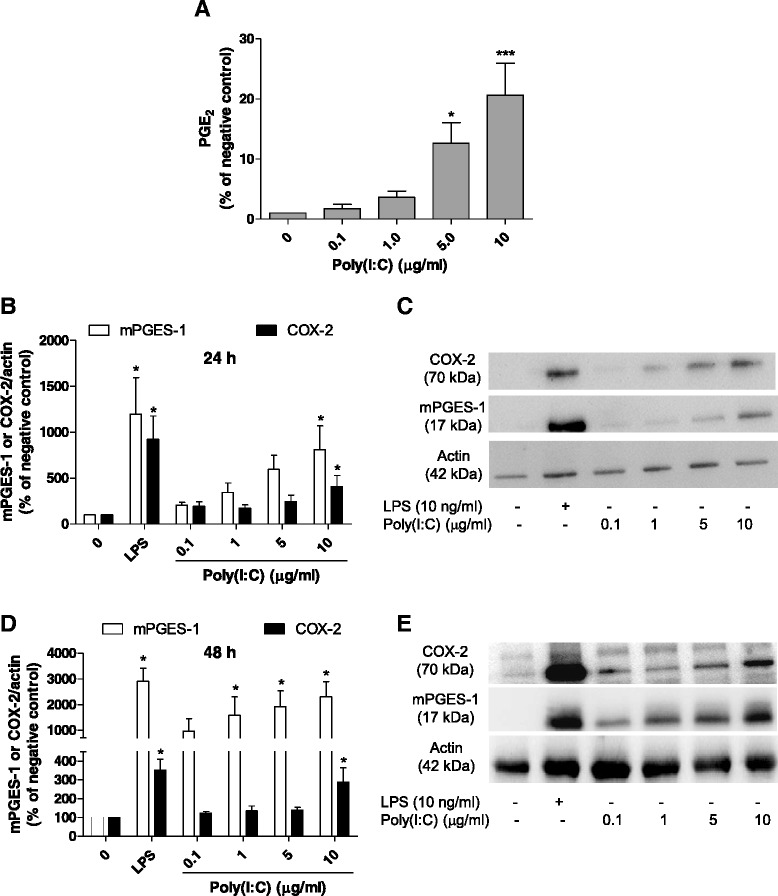
Effect of poly(I:C) on the production of PGE_2_, and mPGES-1 and COX-2 protein levels in primary microglia. **a** Effect of poly(I:C) (0.1–10 μg/ml) on PGE_2_ production after 24 h of stimulation in rat primary microglia. **b**, **d** Quantitative densitometric analysis of COX-2 and mPGES-1 protein levels normalized to actin loading control at 24 (**b**) and 48 h (**d**). **c**, **e** Immunoblot analysis of protein levels of COX-2, mPGES-1, and actin in poly(I:C)-activated microglia at 24 (**c**) and 48 h (**e**). *P < 0.05 and ***P < 0.001 with respect to negative control

We first evaluated the effect of poly(I:C) on the production of PGE_2_, a prostanoid involved in neuroinflammatory conditions. Poly(I:C) increased the production of PGE_2_ at concentrations of 5 and 10 μg/ml in rat primary microglia (Fig. 1a; *P* < 0.05 and 0.001, respectively). In order to investigate the mechanism by which poly(I:C) increases PGE_2_, we investigated the synthesis of mPGES-1 and COX-2 after treatment with poly(I:C). 10 μg/ml of poly(I:C) increased the synthesis of mPGES-1 and COX-2 proteins at 24 (Fig. 1b, c; *P* < 0.05)- and 48-h (Fig. 1d, e; *P* < 0.05) post-stimulation. Interestingly, poly(I:C) increased mPGES-1 synthesis even at lower concentrations (1–10 μg/ml, Fig. 1d, e, *P* < 0.05) at 48 h. In the same conditions, LPS (10 ng/ml), used as a positive control, also increased the synthesis of mPGES-1 and COX-2 (Fig. 1b–e).

### Inhibition of IKK-2, MEK, JNK, and p38 MAPK reduces the expression of mPGES-1 and COX-2 induced by poly(I:C)

**Fig. 2:**
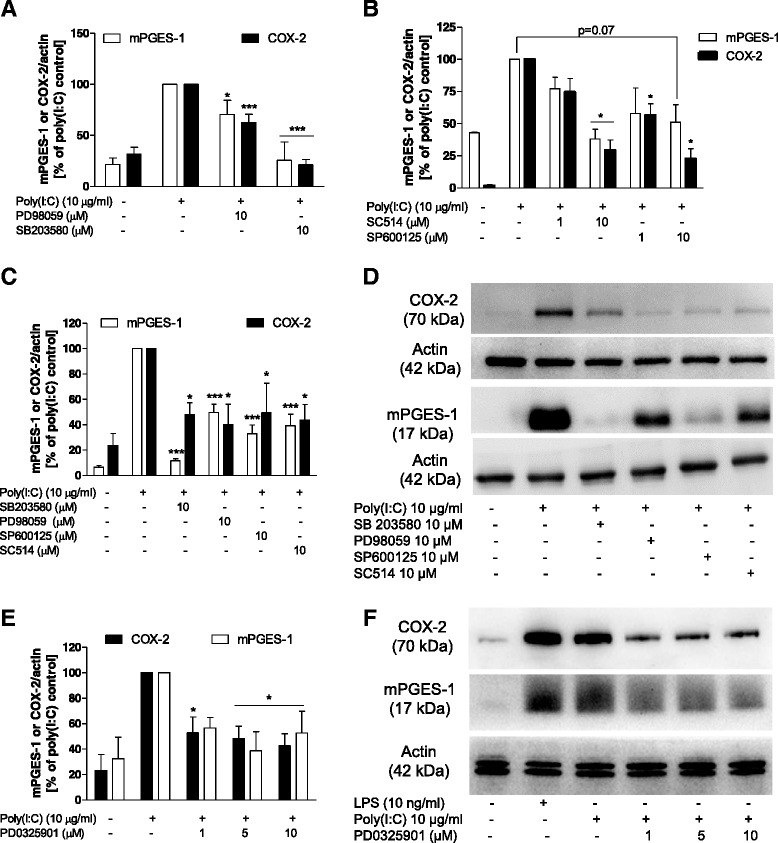
Effect of MEK, MAPK or IKK inhibitors on the protein levels of mPGES-1 and COX-2 at 24 and 48 h after stimulation with poly(I:C) in microglia. **a**, **b** Effect of PD98059 and SB203580 (MEK and p38 MAPK inhibitors, respectively (**a**)) and SP600125 and SC514 (JNK and IKK2 inhibitors, respectively (**b**)) on the protein levels of mPGES-1, COX-2, and actin 24 h after poly(I:C) stimulation. **c, e** Effect of PD98059, SB203580, SP600125, SC514, and PD0325901 (MEK inhibitor) on the protein levels of mPGES-1, COX-2, and actin levels 48 h after poly(I:C) stimulation. **d**, **f** Immunoblot analysis of protein levels of COX-2, mPGES-1, and actin in poly(I:C)-activated microglia 48 h after stimulation. *P < 0.05 and ***P < 0.001 in comparison with the respective poly(I:C) control

As described in our previous studies, mPGES-1 and COX-2 are regulated in microglia by various signal transduction pathways [29, 33] such as *protein kinase C*, *NF-κB*, *MEK*, *JNK*, and *p38 MAPK*. We therefore evaluated whether the increase in COX-2 and mPGES-1 induced by poly(I:C) could be altered by the inhibition of different kinases. As shown in Fig. 2a, b, inhibition of IKK-2, JNK, MEK, and p38 MAPK with SC514, SP600125, PD98059, and SB203580, respectively, reduced the levels of mPGES-1 and COX-2 proteins 24 h after stimulation with poly(I:C) (*P* < 0.05). A similar pattern of inhibition of these kinases was also observed 48 h after stimulation (Fig. 2c, d; *P* < 0.05). Considering that PD98059 might have direct inhibitory effects on COX-2 [34], we evaluated the effect of other MEK inhibitor on the expression of this enzyme, as well as on mPGES-1. We confirmed that PD0325901, a selective MEK1/2 inhibitor, reduced COX-2 and mPGES-1 protein levels induced by poly(I:C) (*P* < 0.05; Fig. 2e, f).

### Inhibition of PI3K and mTOR differently regulates the expression of mPGES-1 and COX-2 induced by poly(I:C)

**Fig. 3:**
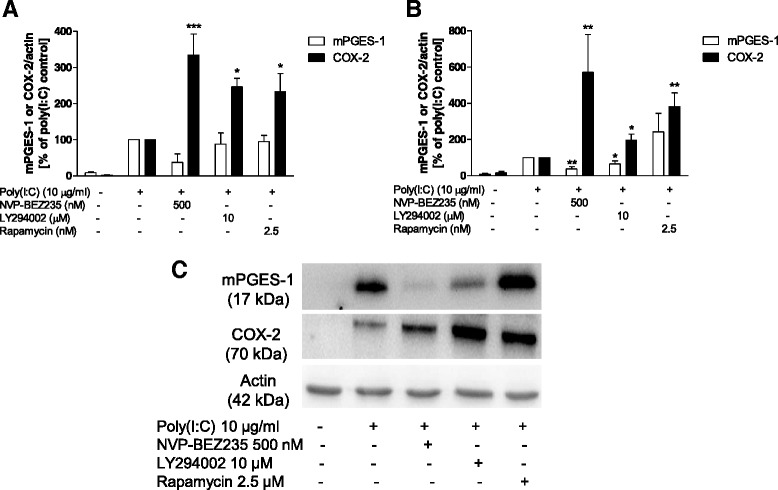
Effect of PI3K or mTOR inhibitors on the protein levels of mPGES-1 and COX-2 at 24 and 48 h after stimulation with poly(I:C) in microglia. **a**, **b** Effect of LY294002, rapamycin, or NVP-BEZ235 (PI3K, mTOR, or PI3K/mTOR dual inhibitors, respectively) on the protein levels of mPGES-1, COX-2, and actin at 24 h (**a**) or 48 h (**b**) after poly(I:C) stimulation. **c** Immunoblot analysis of protein levels of COX-2, mPGES-1, and actin in poly(I:C)-activated microglia 48 h after stimulation. *P < 0.05, **P < 0.01, and ***P < 0.001 in comparison with the respective poly(I:C) control

We have previously demonstrated that inhibition of PI3K and mTOR differently regulates the expression of mPGES-1 and COX-2 in LPS-stimulated rat primary microglia [29, 33]. Thus, we decided to investigate the effect of PI3K and mTOR inhibition on microglia stimulated with a TLR3 agonist. As observed in Fig. 3a–c, LY294002, a PI3K inhibitor, increased the immunoreactivity of COX-2 at 24 and 48 h after stimulation (*P* < 0.001 and *P* < 0.05, respectively), although mPGES-1 was reduced only at 48 h (*P* < 0.05). A similar pattern of the modulation of production of COX-2 and mPGES-1 was also obtained with NVP-BEZ235, a PI3K dual inhibitor (Fig. 3a–c, *P* < 0.05). On the other hand, inhibition of mTOR with rapamycin enhanced the immunoreactivity of COX-2 at 24 and 48 h, although a strong tendency towards induction of mPGES-1 was observed at 48 h after stimulation with poly(I:C) (*P* < 0.05; Fig. 3b, c).

### Poly(I:C) increases the phosphorylation of ERK, JNK, p38 MAPK, Akt, and IκB-α

**Fig. 4:**
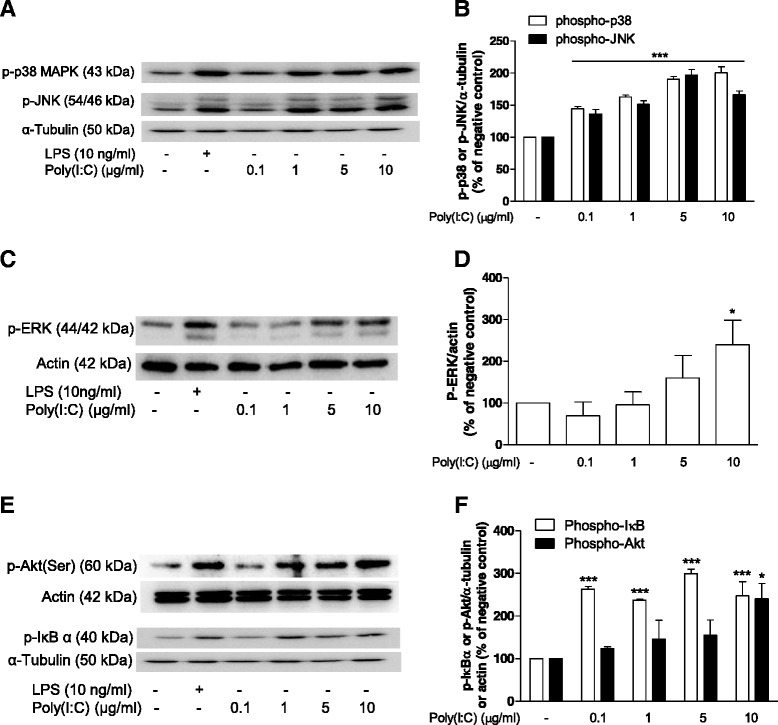
Effect of poly(I:C) on the phosphorylation pattern of intracellular molecules. Representative immunoblot and quantitative analysis of the effects of poly(I:C) on the phosphorylation of p38 and JNK (**a**, **b**), ERK (**c**, **d**), and Akt and IκB (**e**, **f**) 30 min after stimulation. Actin or tubulin was used as controls. *P < 0.05 and ***P < 0.001 in comparison with the respective poly(I:C) control

We next studied whether poly(I:C) directly activated the intracellular signaling molecules described above. Thirty minutes after stimulation, poly(I:C) increased the phosphorylation of JNK and p38 MAPK in all concentrations used (Fig. 4a, b, *P* < 0.001). Phosphorylation of IκB-α were also obtained with the same concentrations (0.1–10 μg/ml; Fig. 4e, f, *P* < 0.001). ERK (Fig. 4c, d, *P* < 0.05) and protein kinase B (Akt) (Fig. 4e, f, *P* < 0.001) were activated by poly(I:C) only at a higher concentration (10 μg/ml). As a control for the experiments, we demonstrated that LPS (10 ng/ml) increased the phosphorylation of IκB-α and all kinases studied (Fig. 4a, c, e) at the same time point.

## Discussion

In the present study, we evaluated the effects of the TLR3 ligand poly(I:C) on the synthesis of enzymes of the arachidonic acid cascade, as well as the intracellular signaling pathways involved in their expressions in rat primary microglia. We demonstrate here that poly(I:C) increases the synthesis of COX-2 and mPGES-1 in rat primary microglia and that these effects are dependent on the activation of MAPKs, PI3K, mTOR, and NF-κB.

Various studies have been performed to elucidate the roles of both mPGES-1 and COX-2, since these enzymes are induced during inflammatory processes. The increased production of PGE_2_ by microglia during neuroinflammatory processes might be important for the development of psychiatric and neurodegenerative processes [22, 35, 36]. Thus, we decided to investigate the effect of a TLR3 agonist on the production of these molecules of the arachidonic acid cascade in microglia. Importantly, poly(I:C) might be considered as an alternative stimulus to LPS to activate microglial cells.

We first demonstrated that poly(I:C) increased the immunoreactivity of both COX-2 and mPGES-1, as well as the production of PGE_2_. In contrast to our results, Gutierrez-Venegas and Rodriguez-Perez [37] demonstrated that poly(I:C) reduced the expression of COX-2 and the production of PGE_2_ induced by histamine in human gingival fibroblasts. These differences observed in the two studies might be due to the different cell type and stimulation agents used. However, another study has shown that, in RAW264.7 cells, poly(I:C) increased the synthesis of COX-2 and PGE_2_ production via TLR3 [38]. Besides, in microglia, poly(I:C) increased the production of TNF-α, IL-6, and IFN-β in a TLR3-dependent manner [28]. In accordance with our data, it has been shown that poly(I:C) increases the expression of mPGES-1 and COX-2 in mouse glial cells [39–41]. However, in these studies, the authors did not investigate whether the effects of poly(I:C) in microglia were mediated by TLR3 and did not elucidate the signaling cascades involved in their syntheses. The increased expression of inflammatory mediators, such as cytokines and prostanoids, induced by dsRNA stimulation, could contribute to the development of the neuropathological processes promoted by the activation of some cytosolic PRRs.

In order to investigate the signaling pathways involved in the synthesis of mPGES-1 and COX-2, we evaluated the effect of poly(I:C) in the phosphorylation of kinases and IκB-α. We demonstrated here that poly(I:C) increased the activation of all MAPKs. These results are in accordance with the study of Steer et al. [42], which demonstrated that poly(I:C) induces MAPK activation in macrophages. Jing et al. [43] showed that activation of TLR3 recruits the complex formed by TRAF6 (TNF receptor-associated factor 6)-TAK1 (TGF-β-activated kinase 1)-TAB2 (TAK1-binding protein 2), which thereafter translocates to the cytosol and interacts with dsRNA-dependent protein kinase (PKR), inducing TAK1 activation. This activation leads to further activation of MAPK and NF-κB. This work was corroborated by another study, which showed that poly(I:C) induced the phosphorylation of MKK3/6 and p38 MAPK in human natural killer cells [44], MKK4/7, and JNK in fibroblast-like synoviocytes [45] and ERK in bone marrow-derived macrophages [46]. However, in human dendritic cells, poly(I:C) failed to induce phosphorylation of JNK, ERK, and p38 MAPK, albeit this effect was observed in rheumatoid arthritis synovial fibroblasts [4]. In accordance with our data, activation of these kinases has also been demonstrated in mouse microglia although the concentrations of poly(I:C) necessary for these effects were higher [28].

MAPKs are important regulators of the expressions of inflammatory mediators. Activation of ERK by poly(I:C) controls the production of nitric oxide and IL-1-β in macrophages [47, 48]. Inhibitors of JNK and p38 MAPK reduced the production of CXCL10 in a human bronchial epithelial cell line and human natural killer cells stimulated with poly(I:C), respectively [44, 49]. JNK and p38 MAPK induce the transcription of genes via activation of different downstream molecules [50, 51]. Moreover, different studies have demonstrated that JNK and p38 MAPK stabilize mRNA, leading to an enhanced transduction. In monocyte-derived dendritic cells, poly(I:C) induced stabilization of IFN-β mRNA, and this effect is mediated by TRIF, MK2, and p38 MAPK [52]. Considering that JNK and p38 MAPK might be involved in the stabilization of COX-2 mRNA [53–55], it is possible that, in our conditions, the reduction in COX-2 observed with the incubation of the cells with JNK and p38 MAPK inhibitors might be due to a reduced stability of the COX-2 mRNA, although a direct effect on gene transcriptions cannot be discarded.

The NF-κB is involved in the expression of different inflammatory mediators. We and others have previously demonstrated that NF-κB regulates the expression of COX-2 and mPGES-1 induced by LPS [33, 56]. However, it has been demonstrated that poly(I:C) and LPS induce distinct NF-κB signaling [57]. Thus, we aimed to investigate whether poly(I:C) induces NF-κB activation in microglia. We have demonstrated that poly(I:C) increased IκB phosphorylation and that SC514, a IKK-2 inhibitor, reduced the expression of both COX-2 and mPGES-1. Thus, we were able to attest that NF-κB is involved in the expression of COX-2 and mPGES-1 induced by poly(I:C). In contrast to our previous results, SC-514 abolished the expression of COX-2 induced by poly(I:C), albeit higher concentrations of SC-514 only partially reduced the expression of this enzyme induced by LPS in microglia [33]. Thus, it might be possible that the mechanism or the kinetics of activation of NF-κB induced by LPS and poly(I:C) differ.

**Fig. 5:**
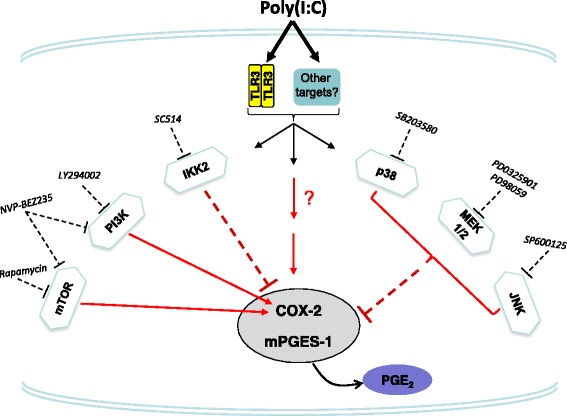
Signal transduction pathways involved in the poly(I:C)-mediated induction of COX-2 and mPGES-1 in rat primary microglia. It is shown here that poly(I:C) induced COX-2 and mPGES-1 proteins, as well as PGE_2_ production, and by using pharmacological inhibitors against various signaling pathways including p38 MAPK, JNK, MEK1/2, and IKK2, these poly(I:C) effects on COX-2 and mPGES-1 were reversed. Furthermore, inhibition of mTOR and PI3K or dual inhibition of PI3K/mTOR enhanced the immunoreactivity of COX-2

The PI3K/Akt/mTOR pathway controls a wide range of physiological and pathological events. These enzymes are involved in the production of inflammatory mediators. Here, we showed that inhibition of mTOR with rapamycin enhanced the synthesis of COX-2. On the other hand, inhibition of PI3K or a dual inhibition of PI3K and mTOR increased the expression of COX-2, albeit the expression of mPGES-1 was partially reduced only at 48 h. We have previously demonstrated that inhibition of PI3K or dual inhibition of PI3K/mTOR increased the expression of COX-2 and reduced the expression of mPGES-1 in LPS-activated microglia [29]. Importantly, Akt is downstream of TRIF and TANK-binding kinase 1 and regulates the expression of IFN-β mRNA in RAW264.7 cells induced by poly(I:C) through interferon regulatory factor 3 (IRF3) activation [58]. Interestingly, Tarassishin and colleagues [59] have recently shown that IRF3 also activates Akt, and inhibition of PI3K with LY294002 reduced the expression of the immunoregulatory cytokines IL-10, IL-1ra, and IFN-β, suggesting an anti-inflammatory role of this kinase. Figure 5 summarizes all the intracellular molecules investigated in this study that contribute to the regulation of COX-2 and mPGES-1 protein levels induced by poly(I:C) in rat primary microglia.

We have previously demonstrated that LPS, a TLR4 ligand, increased the expression of COX-2 and mPGES-1 in primary microglia, and different kinases were involved in the regulation of these two enzymes [29, 33]. Whereas activation of TLR4 utilizes both MyD88 and TRIF, TLR3 signals only via TRIF [7]. Although TLR3 does not stimulate signals via the MyD88 adapter molecule, the signals promoted from TLR3 and TLR4 converge to TRIF, which could lead to further activation of different downstream molecules necessary for COX-2 and mPGES-1 expression.

In the present study, we were not able to prove that the effects shown by poly(I:C) are mediated by the TLR3 in our rat microglia model. It might also be possible that the effects of poly(I:C) are mediated via a TLR3-independent pathway. For example, it has been demonstrated that the effects of poly(I:C) on the expression of COX-2 were not dependent on TLR3 [42]. Furthermore, deficiency of TLR3 also did not avoid the CD8 T cell expansion induced by poly(I:C) [60]. In this same study, it was demonstrated that injection of poly(I:C) increased the levels of serum IL-6 in WT and TLR3^−/−^ mice, albeit the levels of TNF-α were reduced, demonstrating a differential regulation of poly(I:C) on both cytokines [60].

Poly(I:C) might have other targets, such as the melanoma differentiation-associated gene 5-deficient mice (MDA5), which is a cytosolic PRR that recognizes viral RNA. It has been shown that in MDA5^−/−^ mice, poly(I:C) administration did not increase the sera levels of IFN-γ as compared with those in WT mice. The production of IL-6 and IL-12p40 was also impaired in MDA5^−/−^ mice [61]. Moreover, poly(I:C) may also activate the NLRP3 inflammasome in TLR3- and MDA5-independent pathways [62].

## Conclusions

In conclusion, we provide evidences that the signaling cascades involved in the expression of COX-2 and mPGES-1 induced by poly(I:C) are similar with the pathways induced by LPS in microglia. Finally, considering that activation of PRRs, such as TLR3, might be associated with pain and psychiatric and neurodegenerative diseases [2, 9–11], it could be speculated that COX-2, mPGES-1, and PGE_2_ induced by this stimulus could partially contribute to the development of neuropathological conditions.

## Abbreviations

IKK: IκB kinase
Akt: protein kinase B
COX: cyclooxygenase
ERK: extracellular signal-regulated kinase
GSK-3: glycogen synthase kinase-3
IL: interleukin
JNK: c-Jun N-terminal kinase
LPS: lipopolysaccharide
mPGES: microsomal prostaglandin E synthase
MAPK: mitogen-activated protein kinase
MEK: mitogen-activated protein kinase kinase
mTOR: mammalian target of rapamycin
poly(I:C): polyinosinic-polycytidylic acid
PG: prostaglandin
PI3K: phosphatidylinositol 3-kinase
TLR: toll-like receptor
TNF: tumor necrosis factor

## References

[1] Forrest CM, Khalil OS, Pisar M, Smith RA, Darlington LG, Stone TW (2012). Prenatal activation of toll-like receptors-3 by administration of the viral mimetic poly(I:C) changes synaptic proteins, N-methyl-D-aspartate receptors and neurogenesis markers in offspring. Mol Brain.

[2] Reisinger S, Khan D, Kong E, Berger A, Pollak A, Pollak DD (2015). The poly(I:C)-induced maternal immune activation model in preclinical neuropsychiatric drug discovery. Pharmacol Ther.

[3] Kariko K, Ni H, Capodici J, Lamphier M, Weissman D (2004). mRNA is an endogenous ligand for toll-like receptor 3. J Biol Chem.

[4] Lundberg AM, Drexler SK, Monaco C, Williams LM, Sacre SM, Feldmann M (2007). Key differences in TLR3/poly I:C signaling and cytokine induction by human primary cells: a phenomenon absent from murine cell systems. Blood.

[5] Olson JK, Miller SD (2004). Microglia initiate central nervous system innate and adaptive immune responses through multiple TLRs. J Immunol.

[6] Marshall-Clarke S, Downes JE, Haga IR, Bowie AG, Borrow P, Pennock JL (2007). Polyinosinic acid is a ligand for toll-like receptor 3. J Biol Chem.

[7] Kawai T, Akira S (2011). Toll-like receptors and their crosstalk with other innate receptors in infection and immunity. Immunity.

[8] Ribes S, Adam N, Ebert S, Regen T, Bunkowski S, Hanisch UK (2010). The viral TLR3 agonist poly(I:C) stimulates phagocytosis and intracellular killing of Escherichia coli by microglial cells. Neurosci Lett.

[9] Field R, Campion S, Warren C, Murray C, Cunningham C (2010). Systemic challenge with the TLR3 agonist poly I:C induces amplified IFNalpha/beta and IL-1beta responses in the diseased brain and exacerbates chronic neurodegeneration. Brain Behav Immun.

[10] Liu T, Gao YJ, Ji RR (2012). Emerging role of toll-like receptors in the control of pain and itch. Neurosci Bull.

[11] Qian NS, Liao YH, Feng QX, Tang Y, Dou KF, Tao KS (2011). Spinal toll like receptor 3 is involved in chronic pancreatitis-induced mechanical allodynia of rat. Mol Pain.

[12] Stokes JA, Corr M, Yaksh TL (2013). Spinal toll-like receptor signaling and nociceptive processing: regulatory balance between TIRAP and TRIF cascades mediated by TNF and IFNbeta. Pain.

[13] Bobyn J, Mangano EN, Gandhi A, Nelson E, Moloney K, Clarke M (2012). Viral-toxin interactions and Parkinson's disease: poly I:C priming enhanced the neurodegenerative effects of paraquat. J Neuroinflammation.

[14] Deleidi M, Hallett PJ, Koprich JB, Chung CY, Isacson O (2010). The toll-like receptor-3 agonist polyinosinic:polycytidylic acid triggers nigrostriatal dopaminergic degeneration. J Neurosci.

[15] Ribeiro BM, do Carmo MR, Freire RS, Rocha NF, Borella VC, de Menezes AT (2013). Evidences for a progressive microglial activation and increase in iNOS expression in rats submitted to a neurodevelopmental model of schizophrenia: reversal by clozapine. Schizophr Res.

[16] Ozawa K, Hashimoto K, Kishimoto T, Shimizu E, Ishikura H, Iyo M (2006). Immune activation during pregnancy in mice leads to dopaminergic hyperfunction and cognitive impairment in the offspring: a neurodevelopmental animal model of schizophrenia. Biol Psychiatry.

[17] Kato H, Takeuchi O, Mikamo-Satoh E, Hirai R, Kawai T, Matsushita K (2008). Length-dependent recognition of double-stranded ribonucleic acids by retinoic acid-inducible gene-I and melanoma differentiation-associated gene 5. J Exp Med.

[18] Liu J, Guo YM, Hirokawa M, Iwamoto K, Ubukawa K, Michishita Y (2012). A synthetic double-stranded RNA, poly I:C, induces a rapid apoptosis of human CD34(+) cells. Exp Hematol.

[19] de Rivero Vaccari JP, Brand FJ, Sedaghat C, Mash DC, Dietrich WD, Keane RW (2014). RIG-1 receptor expression in the pathology of Alzheimer's disease. J Neuroinflammation.

[20] Huang Y, Halliday GM (2012). Aspects of innate immunity and Parkinson's disease. Front Pharmacol.

[21] Okun E, Griffioen KJ, Lathia JD, Tang SC, Mattson MP, Arumugam TV (2009). Toll-like receptors in neurodegeneration. Brain Res Rev.

[22] Muller N, Myint AM, Schwarz MJ (2012). Immunological treatment options for schizophrenia. Curr Pharm Biotechnol.

[23] O'Banion MK (1999). COX-2 and Alzheimer's disease: potential roles in inflammation and neurodegeneration. Expert Opin Investig Drugs.

[24] Teismann P, Ferger B (2001). Inhibition of the cyclooxygenase isoenzymes COX-1 and COX-2 provide neuroprotection in the MPTP-mouse model of Parkinson's disease. Synapse.

[25] Lima IV, Bastos LF, Limborco-Filho M, Fiebich BL, de Oliveira AC (2012). Role of prostaglandins in neuroinflammatory and neurodegenerative diseases. Mediators Inflamm.

[26] Ricciotti E, FitzGerald GA (2011). Prostaglandins and inflammation. Arterioscler Thromb Vasc Biol.

[27] Suh HS, Zhao ML, Choi N, Belbin TJ, Brosnan CF, Lee SC (2009). TLR3 and TLR4 are innate antiviral immune receptors in human microglia: role of IRF3 in modulating antiviral and inflammatory response in the CNS. Virology.

[28] Town T, Jeng D, Alexopoulou L, Tan J, Flavell RA (2006). Microglia recognize double-stranded RNA via TLR3. J Immunol.

[29] de Oliveira AC, Candelario-Jalil E, Langbein J, Wendeburg L, Bhatia HS, Schlachetzki JC (2012). Pharmacological inhibition of Akt and downstream pathways modulates the expression of COX-2 and mPGES-1 in activated microglia. J Neuroinflammation.

[30] Seregi A, Keller M, Jackisch R, Hertting G (1984). Comparison of the prostanoid synthesizing capacity in homogenates from primary neuronal and astroglial cell cultures. Biochem Pharmacol.

[31] Laemmli UK (1970). Cleavage of structural proteins during the assembly of the head of bacteriophage T4. Nature.

[32] Singh V, Bhatia HS, Kumar A, de Oliveira AC, Fiebich BL (2014). Histone deacetylase inhibitors valproic acid and sodium butyrate enhance prostaglandins release in lipopolysaccharide-activated primary microglia. Neuroscience.

[33] de Oliveira AC, Candelario-Jalil E, Bhatia HS, Lieb K, Hull M, Fiebich BL (2008). Regulation of prostaglandin E2 synthase expression in activated primary rat microglia: evidence for uncoupled regulation of mPGES-1 and COX-2. Glia.

[34] Borsch-Haubold AG, Pasquet S, Watson SP (1998). Direct inhibition of cyclooxygenase-1 and -2 by the kinase inhibitors SB 203580 and PD 98059. SB 203580 also inhibits thromboxane synthase. J Biol Chem.

[35] Block ML, Hong JS (2005). Microglia and inflammation-mediated neurodegeneration: multiple triggers with a common mechanism. Prog Neurobiol.

[36] Wang T, Pei Z, Zhang W, Liu B, Langenbach R, Lee C (2005). MPP + -induced COX-2 activation and subsequent dopaminergic neurodegeneration. FASEB J.

[37] Gutierrez-Venegas G, Rodriguez-Perez CE (2012). Toll-like receptor 3 activation promotes desensitization of histamine response in human gingival fibroblasts: poly (I:C) induces histamine receptor desensitization in human gingival fibroblasts. Cell Immunol.

[38] Pindado J, Balsinde J, Balboa MA (2007). TLR3-dependent induction of nitric oxide synthase in RAW 264.7 macrophage-like cells via a cytosolic phospholipase A2/cyclooxygenase-2 pathway. J Immunol.

[39] Li X, Cudaback E, Keene CD, Breyer RM, Montine TJ (2011). Suppressed microglial E prostanoid receptor 1 signaling selectively reduces tumor necrosis factor alpha and interleukin 6 secretion from toll-like receptor 3 activation. Glia.

[40] Li X, Montine KS, Keene CD, Montine TJ (2015). Different mechanisms of apolipoprotein E isoform-dependent modulation of prostaglandin E2 production and triggering receptor expressed on myeloid cells 2 (TREM2) expression after innate immune activation of microglia. FASEB J.

[41] Li X, Cudaback E, Breyer RM, Montine KS, Keene CD, Montine TJ (2012). Eicosanoid receptor subtype-mediated opposing regulation of TLR-stimulated expression of astrocyte glial-derived neurotrophic factor. FASEB J.

[42] Steer SA, Moran JM, Christmann BS, Maggi LB, Corbett JA (2006). Role of MAPK in the regulation of double-stranded RNA- and encephalomyocarditis virus-induced cyclooxygenase-2 expression by macrophages. J Immunol.

[43] Jiang Z, Zamanian-Daryoush M, Nie H, Silva AM, Williams BR, Li X (2003). Poly(I-C)-induced Toll-like receptor 3 (TLR3)-mediated activation of NFkappa B and MAP kinase is through an interleukin-1 receptor-associated kinase (IRAK)-independent pathway employing the signaling components TLR3-TRAF6-TAK1-TAB2-PKR. J Biol Chem.

[44] Pisegna S, Pirozzi G, Piccoli M, Frati L, Santoni A, Palmieri G (2004). p38 MAPK activation controls the TLR3-mediated up-regulation of cytotoxicity and cytokine production in human NK cells. Blood.

[45] Yoshizawa T, Hammaker D, Sweeney SE, Boyle DL, Firestein GS (2008). Synoviocyte innate immune responses: I. Differential regulation of interferon responses and the JNK pathway by MAPK kinases. J Immunol.

[46] Pauleau AL, Murray PJ (2003). Role of nod2 in the response of macrophages to toll-like receptor agonists. Mol Cell Biol.

[47] Moore TC, Petro TM (2013). IRF3 and ERK MAP-kinases control nitric oxide production from macrophages in response to poly-I:C. FEBS Lett.

[48] Maggi LB, Moran JM, Buller RM, Corbett JA (2003). ERK activation is required for double-stranded RNA- and virus-induced interleukin-1 expression by macrophages. J Biol Chem.

[49] Lam KP, Chu YT, Kuo CH, Wang WL, Tok TS, Chin YY (2011). Suppressive effects of procaterol on expression of IP-10/CXCL 10 and RANTES/CCL 5 by bronchial epithelial cells. Inflammation.

[50] Zarubin T, Han J (2005). Activation and signaling of the p38 MAP kinase pathway. Cell Res.

[51] Waetzig V, Czeloth K, Hidding U, Mielke K, Kanzow M, Brecht S (2005). c-Jun N-terminal kinases (JNKs) mediate pro-inflammatory actions of microglia. Glia.

[52] Johnsen IB, Nguyen TT, Bergstrom B, Lien E, Anthonsen MW (2012). Toll-like receptor 3-elicited MAPK activation induces stabilization of interferon-beta mRNA. Cytokine.

[53] Dean JL, Brook M, Clark AR, Saklatvala J (1999). p38 mitogen-activated protein kinase regulates cyclooxygenase-2 mRNA stability and transcription in lipopolysaccharide-treated human monocytes. J Biol Chem.

[54] Lasa M, Mahtani KR, Finch A, Brewer G, Saklatvala J, Clark AR (2000). Regulation of cyclooxygenase 2 mRNA stability by the mitogen-activated protein kinase p38 signaling cascade. Mol Cell Biol.

[55] Nieminen R, Lahti A, Jalonen U, Kankaanranta H, Moilanen E (2006). JNK inhibitor SP600125 reduces COX-2 expression by attenuating mRNA in activated murine J774 macrophages. Int Immunopharmacol.

[56] D'Acquisto F, Iuvone T, Rombola L, Sautebin L, Di Rosa M, Carnuccio R (1997). Involvement of NF-kappaB in the regulation of cyclooxygenase-2 protein expression in LPS-stimulated J774 macrophages. FEBS Lett.

[57] Reimer T, Brcic M, Schweizer M, Jungi TW (2008). poly(I:C) and LPS induce distinct IRF3 and NF-kappaB signaling during type-I IFN and TNF responses in human macrophages. J Leukoc Biol.

[58] Joung SM, Park ZY, Rani S, Takeuchi O, Akira S, Lee JY (2011). Akt contributes to activation of the TRIF-dependent signaling pathways of TLRs by interacting with TANK-binding kinase 1. J Immunol.

[59] Tarassishin L, Suh HS, Lee SC (2011). Interferon regulatory factor 3 plays an anti-inflammatory role in microglia by activating the PI3K/Akt pathway. J Neuroinflammation.

[60] Ngoi SM, Tovey MG, Vella AT (2008). Targeting poly(I:C) to the TLR3-independent pathway boosts effector CD8 T cell differentiation through IFN-alpha/beta. J Immunol.

[61] Kato H, Takeuchi O, Sato S, Yoneyama M, Yamamoto M, Matsui K (2006). Differential roles of MDA5 and RIG-I helicases in the recognition of RNA viruses. Nature.

[62] Rajan JV, Warren SE, Miao EA, Aderem A (2010). Activation of the NLRP3 inflammasome by intracellular poly I:C. FEBS Lett.

